# Beyond Training: Systems Framework for Sustainable Health Informatics Investment in Africa

**DOI:** 10.2196/89482

**Published:** 2026-06-01

**Authors:** Eric Nzirakaindi Ikoona, Lucy Namulemo, Ronald Kaluya, Rebecca Ikoona, Foday Sahr

**Affiliations:** 1 National Public Health Agency, 42A Main Motor Road, Wilberforce, Freetown, Western Area, 232, Sierra Leone, 232 076680085; 2 Uganda Counselling and Support Services, Kampala, Uganda; 3 Foothills Community-Based Interventions, Monticello, KY, United States; 4 Makerere University–Johns Hopkins Research Collaboration, Kampala, Uganda

**Keywords:** digital health, health informatics, health information systems, District Health Information Software 2, DHIS2, workforce development, health systems strengthening, Africa, public health surveillance, artificial intelligence

## Abstract

Across Africa, substantial investment has built national health information systems (HISs), including the surveillance platforms, reporting tools, and digital infrastructure through which health data flow. However, the health informatics capacity needed to sustain those systems remains fragile: trained health informaticians leave for better-resourced organizations, platforms fall into disrepair when donor funding ends, and data systems multiply without connecting. Health informatics is the discipline that designs, governs, and sustains HISs; the two are inseparable, and investment that builds one while neglecting the other cannot produce durable results. This viewpoint, grounded in the authors’ direct implementation experience across sub-Saharan Africa and informed by published literature, argues that the cause is a structurally misaligned investment logic: resources concentrate on training health informaticians while the institutional, governance, and infrastructure conditions that determine whether those informaticians can perform are chronically underfunded. We propose that sustainable health informatics capacity requires a functioning HIS ecosystem and that this ecosystem rests on four interdependent pillars: (1) workforce development beyond training; (2) institutional strengthening; (3) governance and data standards; and (4) interoperable infrastructure, including the national HIS platforms, such as the District Health Information Software 2, through which health informatics practice operates. The pillars are interdependent: weakness in any one undermines the others in specific, predictable ways. The framework makes 3 contributions beyond existing World Health Organization (WHO) and Africa Centers for Disease Control and Prevention frameworks: it shows how weakness in one pillar actively undermines the others; it focuses specifically on health informatics and national HISs as the primary investment domain rather than as components of a broader digital health agenda; and it treats the enabling environment, specifically civil service structures, domestic financing, data sovereignty, and artificial intelligence governance, as core investment requirements rather than background conditions. Drawing on illustrative experiences from Ethiopia, Kenya, and Sierra Leone, we show how health informatics capacity succeeds or fails depending on the strength of all 4 conditions. A practical readiness checklist and audience-specific policy recommendations are provided for national health ministries, national public health institutes, regional bodies, and development partners.

## Introduction

Over the past decade, African governments and development partners have invested substantially in digital health. More than 40 nations reportedly use the District Health Information Software 2 (DHIS2) for routine health information management [1,2], and health workers—from rural facilities to national disease control centers—now use digital platforms such as DHIS2-based surveillance modules, electronic disease notification systems, and mobile reporting tools to track outbreaks, monitor immunization coverage, and coordinate emergency responses [3,4]. The infrastructure investment is real and significant, but the capacity to sustain and use it effectively has not kept pace. This gap, between platforms built and platforms functioning, is the problem this paper addresses.

Understanding why requires a precise distinction between 2 related but different concepts. A health information system (HIS) is the operational infrastructure of a health system—the surveillance platforms, electronic health records, reporting tools, registries, and dataflows through which health data are generated, managed, and used. DHIS2, the most widely deployed HIS platform in Africa, is an example. Health informatics is the discipline that designs, governs, and continuously improves those systems—the trained workforce, institutional arrangements, data standards, interoperability frameworks, and governance rules that determine whether an HIS functions well or poorly. An HIS is the system. Health informatics is what makes the system work. The relationship between them is the paper’s central concern: health informatics capacity must be embedded within a functioning HIS ecosystem, and a functioning HIS ecosystem requires health informatics capacity to sustain it. Investment that treats them as separate problems produces neither.

However, the dominant investment logic does not reflect this relationship. Across Africa, health informatics investment concentrates on training: initiatives such as Africa Centers for Disease Control and Prevention’s (CDC’s) Africa Epidemic Services-Public Health Informatics (AES-PHI) fellowship produce competency-trained graduates and directly address workforce shortages [5,6]. This is necessary, but training produces individuals, not systems. A trained informatician placed in an institution without a funded post, a governance mandate, or functional HIS infrastructure cannot perform. The informatician and the system each need the other, and investment that builds one while neglecting the other cannot produce sustainable health informatics capacity. Existing frameworks recognize that workforce, governance, and infrastructure are all important: the World Health Organization (WHO), the World Bank, and Africa CDC have consistently identified them as interdependent requirements for sustainable health system performance [2,5,7-9], but they treat these elements as parallel components rather than as interdependent conditions whose misalignment is itself the cause of failure. What has been missing is a framework that explains, specifically for health informatics, how misalignment across these conditions limits the translation of training investment into functioning national HISs.

We therefore propose a 4-pillar systems framework for sustainable health informatics investment in Africa, one that goes beyond training to address the full set of conditions a health informatician needs to perform. The 4 pillars are workforce development beyond training, institutional strengthening, governance and data standards, and interoperable infrastructure. The framework is designed for 4 audiences: national health ministries responsible for health informatics investment decisions, national public health institutes (NPHIs) that serve as institutional anchors for surveillance and health informatics practice, regional bodies such as Africa CDC that set standards and coordinate capacity, and development partners whose financing decisions shape what gets built and what gets sustained.

## From Paper to Platform: The African Digital Health Journey

### Overview

For decades, African health systems ran on paper, with data arriving late, incomplete, and largely unused. The 2014 to 2016 Ebola outbreak exposed these gaps and accelerated investment in HISs across the region [1,2]. DHIS2, developed by the Health Information Systems Program at the University of Oslo, became the platform of choice: by 2020, over 40 African countries reportedly had national DHIS2 platforms supporting routine health information, surveillance, or both [1,10,11].

The country experiences mentioned under the *Ethiopia*, *Kenya*, and *Sierra Leone* sections are illustrative, not a systematic review. Their purpose is to show how the relative strength or weakness of specific enabling conditions shapes health informatics capacity. Those conditions map to the 4 pillars of the framework presented in the section that follows.

### Ethiopia

Ethiopia’s nationwide DHIS2 rollout, combined with the electronic community HIS, formed part of a sustained federal investment in digital health embedded in successive national health sector transformation plans [12]. An interrupted time series study subsequently reported improvements in maternal and child health indicators, documenting gains in data completeness and reporting timeliness [13]. In the authors’ assessment, Ethiopia’s trajectory illustrates an uneven pillar profile. Pillars 1 and 4 were relatively strong: the country built substantial informatics workforce capacity and invested in interoperable infrastructure. Pillars 2 and 3 lagged: harmonizing parallel vertical systems required cross-ministry mandates and consolidated data standards that took much longer to establish. The result was a system where skills and infrastructure existed but institutional coordination and governance frameworks were absent. This constrained comprehensive integration. It is a direct illustration of why pillar 4 investment alone cannot compensate for weak pillars 2 and 3.

### Kenya

Kenya offers a contrasting trajectory. The 2011 to 2012 national DHIS2 rollout documented investment across all 4 enabling conditions: informatics workforce with defined institutional roles (pillar 1), coordination structures linking district and national health system levels (pillar 2), frameworks governing data access and use (pillar 3), and the technical infrastructure to connect them (pillar 4) [14]. The result was a more integrated national platform than comparator settings where investment was concentrated on a single domain [14,15]. The contrast with Ethiopia is not about resources, it is about alignment: investing across all 4 conditions simultaneously produced better integration than concentrating on 1 or 2 conditions.

### Sierra Leone: Crisis-Driven Innovation and Systems Learning

Sierra Leone did not plan its way to building health informatics capacity; crisis forced it. The 2014 to 2016 Ebola epidemic exposed acute gaps in surveillance and coordination. It drove rapid investment from the Ministry of Health and Sanitation, the US CDC, WHO, and partners [16,17]. In 2019, Sierra Leone became the first country in the WHO African region to achieve nationwide electronic health facility reporting of surveillance data [16].

The electronic Integrated Disease Surveillance and Response system, built on DHIS2, now operates across all 16 districts and more than 1500 health facilities [16,17]. Completeness of weekly IDSR reports rose from 68% in 2015 to above 98% in 2023, according to national DHIS2 data in the peer-reviewed literature [18]; these figures reflect reported national routine data and should be interpreted accordingly. Four enabling conditions drove this improvement: a trained surveillance workforce, a strengthened National Public Health Agency with a statutory mandate, standardized data definitions and reporting protocols, and a hybrid infrastructure combining SMS text messaging, mobile data entry, and web interfaces to accommodate varied connectivity [16,19].

When the COVID-19 pandemic arrived, Sierra Leone did not build new systems. National teams adapted the existing DHIS2-based platform for surveillance, contact tracing, and laboratory modules [11]. Precrisis investment in platforms and workforce enabled a faster, more coordinated response than would otherwise have been possible.

Two subsequent emergencies further tested this infrastructure. A synthetic cannabinoid crisis (locally called Kush) and concurrent mpox outbreaks [19,20] required rapid multisectoral coordination for which no dedicated parallel system existed. The National Public Health Agency coordinated responses using real-time DHIS2 data [19,20]—no new system was built. This is the clearest demonstration the Sierra Leone experience offers: when all 4 pillars are in place, routine HIS infrastructure can absorb novel threats without emergency workarounds.

## A Systems Framework for Sustainable Health Informatics Investment

### Overview

**Figure 1.**
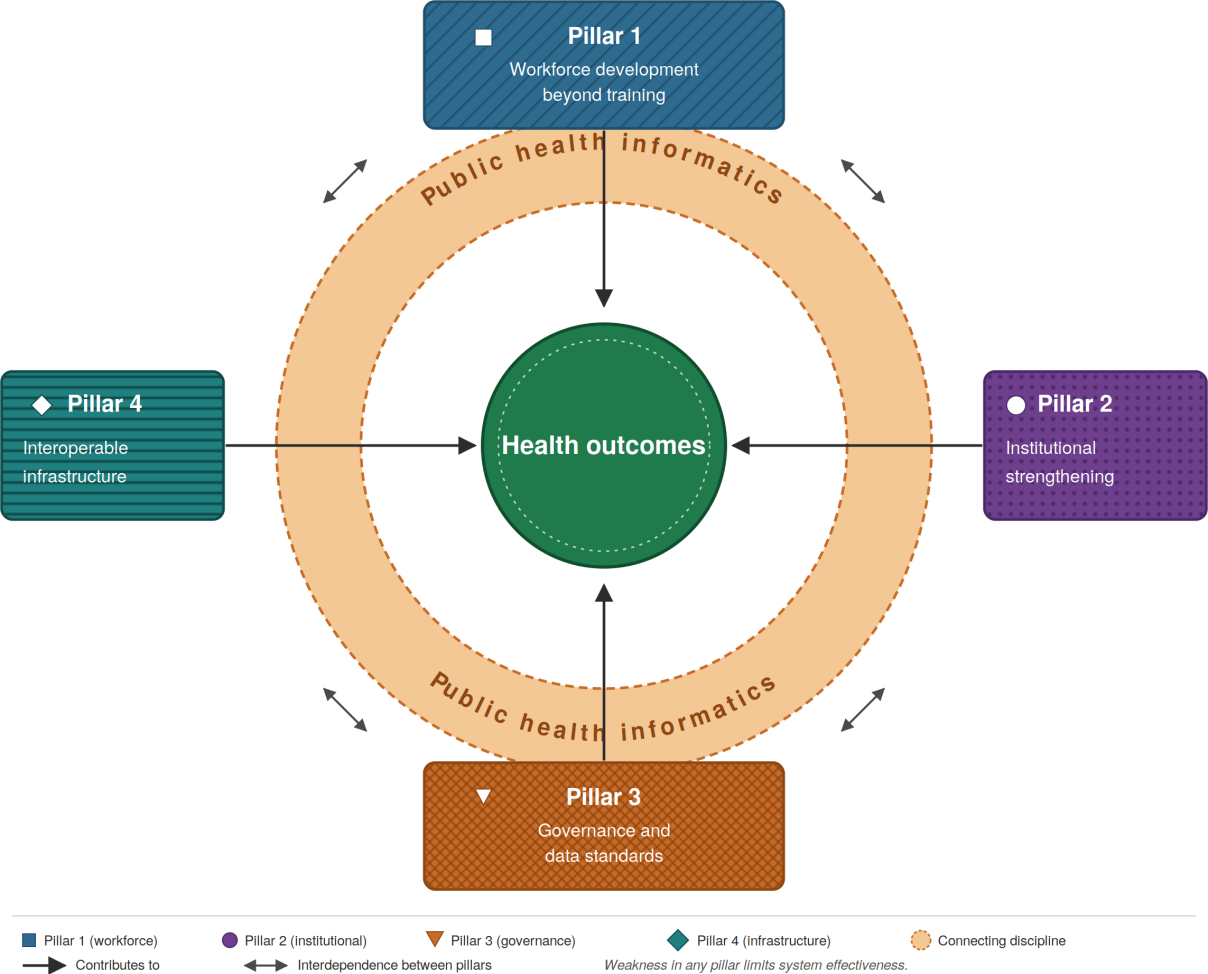
A systems framework for sustainable public health informatics investment in Africa. Four interdependent pillars (workforce development, institutional strengthening, governance and data standards, and interoperable infrastructure) converge on health outcomes. Public health informatics (orange ring) is the connecting discipline. Bidirectional arrows denote interdependence; weakness in any pillar limits system effectiveness.

The country experiences above illustrate what happens when some of these conditions are in place and others are not. The framework shown in Figure 1 makes those conditions explicit and maps their interdependencies. Each pillar is addressed in the subsections below; the failure mechanisms linking them are addressed in the following section.

### Pillar 1: Workforce Development Beyond Training

Health informatics cannot function without trained practitioners. Africa CDC’s workforce framework identifies health informatics as a priority domain [5], and the AES-PHI fellowship is one of the most important mechanisms for building this cadre [6]. However, training is the starting point, not the destination.

A stable workforce requires more than a training certificate. It requires formal civil service career pathways within ministries of health and NPHIs, salary structures that reduce attrition to the private sector and international organizations, structured mentoring, continuing professional development programs, and cross-border learning networks [21].

When these conditions are absent, fellowship graduates can migrate to better-resourced organizations, leaving the institutions that trained them without the human capital required to execute their mandates. This dynamic directly undermines pillar 2: institutions lose their most capable staff before those staff can build the routines and practices that make the institution function.

### Pillar 2: Institutional Strengthening

Trained individuals cannot act without institutions that give them authority, resources, and a mandate. The gap between institutional presence and institutional capacity is well documented. Eighty percent of African countries have established a public health emergency operations center (PHEOC). However, fewer than one-third meet most core functionality criteria [22]. A structure on paper and a functioning institution are not the same thing.

The WHO–Africa CDC PHEOC framework establishes that functional operations centers require coherent policies, defined data systems, and stable skilled staffing working together—not physical infrastructure alone [23]. Clear mandates, standard operating procedures, and stable funding are the conditions under which data and skills can translate into coordinated action. Without them, neither does.

NPHIs are the natural institutional anchors for health informatics practice. They combine a statutory public health mandate with technical independence from line ministries, and can host surveillance systems, PHEOCs, and analytic units under a single roof [21,24]. In many settings, the binding constraint is not talent but the absence of a dedicated domestic budget line. Health informatics work that depends on donor funding cycles rarely aligns with national planning horizons. When grants end, the capacity they supported is at risk.

When institutional strengthening is weak, informaticians trained under pillar 1 can lack the resource authority, legal mandate, and budget to act. Their technical skills risk being stranded. Without an institutional anchor, governance frameworks and data-sharing agreements may remain aspirational: rules without the authority to enforce them.

### Pillar 3: Governance and Data Standards

Data without governance are a liability, not an asset. Across Africa, many countries lack enforceable rules for cross-program data access, data-sharing agreements, and privacy protections [25]. Fragmented vertical systems, each funded by a separate donor project, complicate data harmonization and increase reporting burden on health workers. The WHO Regional Office for Africa identifies governance as a core HIS building block, and these gaps remain pervasive across the region [7]. When standards are absent and governance is unenforced, pillar 4 infrastructure defaults to program-specific specifications. Those specifications cannot be shared across systems, reproducing exactly the fragmentation that coordinated investment was intended to resolve.

The governance imperative extends to artificial intelligence (AI). AI tools for outbreak detection, clinical decision support, and resource allocation are being deployed across African health systems—in many cases ahead of the governance frameworks needed to guide them [26,27]. Without proactive national policies, the risks are concrete: AI data stewardship roles go undefined, consent requirements remain unspecified, and parameters for cross-border data sharing are left to external actors. These tools can then encode existing biases and concentrate analytical control outside the continent. Governance is not a constraint on informatics development. It is what makes that development safe, equitable, and nationally accountable.

### Pillar 4: Interoperable Infrastructure

Infrastructure is not a background condition. It is an active determinant of whether the other three pillars can function. The Open Health Information Exchange architecture provides a widely adopted blueprint, defining components for client registries, facility registries, terminology services, and interoperability layers [28]. Sierra Leone’s Integrated Disease Surveillance and Response experience illustrates how hybrid infrastructure design can accommodate real-world connectivity constraints while feeding a single national platform, combining SMS text messaging, offline data entry, and web interfaces.

Infrastructure investments must address reliable power and connectivity for rural facilities, hardware procurement and maintenance plans, interoperability layers connecting DHIS2 with laboratory and logistics systems, and cybersecurity protections. When infrastructure is unreliable or noninteroperable, health workers can revert to paper, data gaps accumulate, and rural facilities may effectively drop out of national surveillance and resource allocation. The professional confidence of the informatics workforce can erode, reinforcing the attrition that pillar 1 works to prevent.

## Relation to Existing Frameworks and New Contribution

This framework builds on an established conceptual lineage. The WHO health systems building blocks [29] identified six interdependent foundations of functioning health systems: health workforce, health information, financing, medical products, leadership and governance, and service delivery. It has shaped health systems discourse for nearly two decades [29]. The Africa CDC workforce framework [5] and the WHO framework for implementing the global strategy on digital health [7] apply that logic to workforce development and digital health ecosystems respectively. These frameworks establish the consensus that no single investment domain is sufficient. The contribution of this paper is not to identify new components of digital health systems. It is to explain how misalignment across these components limits the translation of health informatics investment into functioning national HISs. None of the existing frameworks specifies how weakness in one domain actively undermines investment in another or which direction that failure travels. None is scoped specifically to health informatics as the primary investment domain, and none treats the enabling environment, specifically civil service structures, domestic funding models, data sovereignty, and AI governance, as core investment requirements rather than supplementary considerations.

Against those three gaps, this framework makes three specific advances. First, it frames health informatics as the primary investment domain and treats HIS platforms as the core infrastructure through which that discipline operates, rather than as one component among many in a broader digital health agenda. Second, it explains how weakness in one pillar undermines the others: an informatician placed in an institution without a mandate, budget, or functional infrastructure cannot perform, regardless of the quality of their training; failure pathways run in both directions and explain why single-pillar investments so frequently underperform. Third, it treats the enabling environment, specifically civil service structures, domestic financing, data sovereignty, and AI governance, as core investment requirements rather than background conditions. Existing frameworks name these as context. This framework treats them as investment objects without which the other pillars cannot be sustained. Table 1 maps these gaps and contributions systematically.

**Table 1.** Comparison of this framework with existing Africa Centers for Disease Control and Prevention (CDC) and World Health Organization (WHO) frameworks.

Feature	WHO building blocks [29]; Africa CDC and WHO digital health frameworks [5,7]	This framework
Core domains identified	Six building blocks: health workforce, health information, financing, medical products, leadership or governance, and service delivery [29]; adapted to digital health as workforce, governance, infrastructure, and human resources [5,7]	Same 4 domains, explicitly named as pillars with defined interdependencies
Failure mechanisms	Building blocks note interdependence but do not map failure pathways; digital health frameworks list domains without interaction logic or sequencing guidance	Bidirectional failure pathways mapped (eg, weak pillar 2 strands pillar 1 investment, and absent pillar 3 fragments pillar 4)
Focus object	Broad digital health ecosystem	HIS^a^ and public health informatics as primary investment objects; interoperability as shared national asset
Political economy	Not addressed	Civil service career pathways, domestic budget lines, and donor-dependency cycles
Data sovereignty and AI^b^ governance	Mentioned generically or absent	Treated as core governance requirements with operational implications for platform hosting and policy review cycles
Equity dimensions	Not systematically addressed	Urban-rural connectivity gaps, gender-stratified access, and frontline worker inclusion in governance
Investment sequencing guidance	Not provided	Readiness checklist enables pillar-by-pillar gap assessment and prioritization

a HIS: health information system.

b AI: artificial intelligence.

How the pillars undermine each other follows predictable patterns. Weak institutional mandates (pillar 2) strand trained informaticians (pillar 1): without a funded post or decision-making authority, a fellowship graduate has no mechanism through which to act. Fragmented data governance (pillar 3) compounds this: absent standards mean workforce competencies trained for one program cannot transfer across systems. Absent governance also fragments infrastructure (pillar 4): platforms built without enforced interoperability standards cannot be connected, reproducing the vertical silos investment was meant to resolve. Moreover, as infrastructure scales, governance must keep pace: AI tools and cross-border dataflows that go unaddressed convert new technical capacity into new institutional risk. The failure runs in reverse too: unreliable infrastructure erodes the working environment of the informatics workforce, reinforcing the attrition that pillar 1 investment works to prevent. These are the patterns through which single-pillar investments so frequently underperform.

As the pillars depend on each other, the order in which they are strengthened matters. When a country trains health informaticians before establishing the institutional structures that give them a post and a mandate, the graduates leave. When it builds HIS infrastructure before putting governance and data standards in place, platforms become program-specific silos that cannot communicate. In low-baseline settings, pillar 2 must come first: without a mandated institution with a budget and defined governance roles, neither workforce retention nor data governance enforcement is achievable. Pillar 3 should precede major pillar 4 investment for the same reason. In higher-baseline settings where pillars 2 and 3 are functional, pillars 1 and 4 can advance together. No sequence is universal: the readiness checklist in Table 2 provides a tool for identifying which pillar is the binding constraint in a given country context.

**Table 2.** Minimum enabling conditions, example indicators, and sequencing priority by pillar. Sequencing priority indicates whether a pillar is a prerequisite for others (must be established first), concurrent (can be developed in parallel), or dependent (requires other pillars to be functional first).

Pillar	Minimum enabling conditions	Example indicators	Sequencing priority
Pillar 1: workforce development	Civil service posts for informaticians; retention incentives; CPD^a^ programs; structured mentoring	Vacancy rate; turnover rate; CPD completion rate; fellowship-to-post conversion rate	Concurrent in high-baseline settings; dependent on pillar 2 in low-baseline settings (no post=no retention)
Pillar 2: institutional strengthening	NPHI^b^ or PHEOC^c^ with statutory mandate; dedicated domestic budget line; clear HIS^d^ governance roles	PHEOC functionality score; budget share for digital health; SOP^e^ coverage for core functions	Prerequisite in most settings. Without mandate and budget, pillars 1, 3, and 4 lack an authorizing home
Pillar 3: governance and data standards	Approved national digital health policy; DSAs^f^; AI^g^ governance principles; interoperability standards	Number of signed DSAs; data breach response time; AI use case registry; proportion of systems using national standards	Prerequisite before major pillar 4 investment. Infrastructure without governance defaults to vertical silos
Pillar 4: interoperable infrastructure	National HIS platform; interoperability layer connecting DHIS2, laboratory, and logistics systems; backup and uptime plan; cybersecurity protections	System uptime (%); API^h^ transactions per month; facility connectivity rate; backup test success rate	Dependent on pillars 2 and 3. In high-baseline settings, simultaneous investment with pillar 1 retention pathways is optimal

a CPD: continuing professional development.

b NPHI: national public health institute.

c PHEOC: public health emergency operations center.

d HIS: health information system.

e SOP: standard operating procedure.

f DSA: data-sharing agreement.

g AI: artificial intelligence.

h API: application programming interface.

## Practical Checklist: Minimum Enabling Conditions by Pillar

Table 2 provides indicative minimum enabling conditions, example indicators, and sequencing priority for each pillar. These are intended as a starting point for government and partner readiness assessments, not as a prescriptive checklist.

## Implementation Challenges, Counterarguments, and Scope

This framework is analytically coherent. Implementing it is another matter. Politically, health ministries and development partners favor visible, countable outputs: training certificates and installed hardware. Systemic investments such as civil service reform and interoperability standards are harder to measure and harder to fund. Financially, sustaining recurring platform costs remains a persistent challenge: the funding landscape for disease surveillance digitalization in Africa is fragmented and predominantly externally driven [30]. Logistically, coordinating across ministries, regulatory bodies, and donor programs demands sustained political will and a named institutional owner with authority to enforce standards.

None of these obstacles is insurmountable. Ministries can build the political case for systemic investments by linking them to outcomes that are already valued: demonstrating that interoperable platforms reduced outbreak response times, for instance, makes the case for infrastructure investment in terms that existing accountability frameworks can register. Governments can pool regional resources to distribute platform maintenance costs and establish domestic budget lines to cofinance core infrastructure, reducing donor dependency over time.

The framework carries inherent limitations. It is grounded in published literature and the authors’ direct implementation experience in a limited set of East and West African countries. Evidence for specific interventions is uneven, implementation contexts vary substantially across the continent, and the framework has not been prospectively validated. Country examples are illustrative; causal claims should not be drawn from them. Future empirical work could test and refine the framework through comparative implementation studies, workforce retention analyses linking civil service post availability to fellowship graduate outcomes, and validation of the pillar-based readiness indicators in Table 2.

A genuine counterargument deserves acknowledgment. Technology-first approaches have achieved real gains: rapid DHIS2 deployment during the Ebola response created surveillance infrastructure that proved adaptable to subsequent crises [11,16,17]. There are settings where building the platform first was more pragmatic than waiting for all enabling conditions to be in place. This framework does not argue against emergency or opportunistic investment. It argues that such investment must be followed, as rapidly as possible, by the institutional and governance conditions that prevent the gains from remaining fragile. Sierra Leone’s experience is illustrative precisely because the crisis-driven infrastructure investment was followed by deliberate strengthening of all 4 pillars.

## Equity and Political-Economy Considerations

Digital health transformation does not occur in a neutral space. Within countries, urban facilities are typically connected before rural and remote clinics, compounding existing inequities in health system access [30,31]. Language barriers and gender norms shape who is recruited into informatics training programs and who benefits from digital tools [2,31]. Without deliberate corrective investment, digital health rollout can widen rather than narrow health system inequity. A system optimized purely for efficiency, connecting well-resourced facilities and training the most accessible graduates, will generate impressive aggregate indicators while leaving the most vulnerable populations underserved.

At the global level, data ownership and sovereignty are actively contested. External partners frequently host data or analytic platforms in servers outside the country, limiting national autonomy and generating well-founded concerns about data extraction [30,31]. Funding patterns that favor short-term pilots over sustained system investment perpetuate fragmentation and external dependency. Treating data sovereignty as a core pillar 3 governance requirement means mandating local hosting, national data stewardship roles, and African-led AI oversight. Development partners committed to African health security should align their platform-hosting and financing decisions accordingly.

## Regional Integration and Cross-Border Collaboration

Infectious diseases do not respect national borders, and neither can HISs. Africa CDC’s coordinating role during the COVID-19 pandemic demonstrated the value of shared platforms and regional institutional capacity [24], and the agency continues to support PHEOC development, cross-border surveillance networks, and common standards for laboratory and surveillance systems [5,21]. Each pillar has a cross-border dimension: regionally coordinated workforce development reduces brain-drain from smaller countries, harmonized governance and data standards lower the costs of cross-border data exchange, and common infrastructure standards allow national platforms to communicate during outbreaks without bespoke interoperability work for each event. These regional dimensions are not optional extensions of the framework. They are the conditions under which the framework can operate at the scale that continental health security requires.

## Policy Implications and Recommendations

Translating the framework into action requires coordinated steps from governments, regional bodies, and development partners.

*National governments* have 4 priority actions. First, establish civil service career tracks for health informaticians with defined job descriptions, promotion pathways, and competitive salaries, as the primary mechanism for reducing postfellowship attrition [5,6,21]. Second, strengthen NPHIs and PHEOCs as institutional anchors for surveillance and digital health coordination, with investment in mandate, budget, and staffing capacity, not structure alone [22]. Third, establish dedicated domestic budget lines for national HIS platforms and infrastructure maintenance, treating interoperability as a shared public good [30]. Fourth, develop or update national digital health strategies to integrate HIS governance, AI oversight, equity indicators, and data sovereignty provisions, reviewed at least every 3 to 5 years [7,8,25].

*Regional bodies* have three priority actions. First, expand training programs in public health informatics with strong placement linkages to NPHIs and PHEOCs, building on the AES-PHI model to ensure graduates enter funded posts rather than unfilled institutional vacancies [5,6,21]. Second, promote harmonized standards for cross-border data exchange, privacy protection, and AI ethics, building on WHO and Open Health Information Exchange guidance [7,25,28]. Third, create peer-learning networks for technical platform maintenance, allowing countries to share infrastructure expertise and avoid duplicating the fixed costs of building national platform capacity independently.

*Development partners* have 4 priority actions. First, shift financing from parallel, program-specific information systems toward long-term support for national platforms and the interoperability standards that connect them [1,11,30]. Second, fund civil service post creation and workforce retention incentives alongside fellowship training. The return on training investment depends entirely on the institutional conditions that allow graduates to remain and act [5,6]. Third, condition infrastructure grants on the prior existence of a governance framework and a named institutional owner, so that pillar 4 investment does not precede the pillar 2 and pillar 3 conditions required to sustain it [23,25]. Fourth, adopt multipillar readiness assessments before designing new digital health programs; the checklist in Table 2 provides a starting framework for identifying which enabling conditions are weakest and sequencing investments accordingly.

## Conclusions

Africa has built something real. Post-Ebola and post–COVID-19 pandemic investments have created a foundation of surveillance systems, PHEOCs, and digital platforms that did not exist a decade ago. The gains are documented and significant, but they are also fragile. Sustaining and expanding them requires a shift not in effort but in logic: the goal is not to produce trained individuals but to build the enabling ecosystems in which those individuals can act.

What makes the framework useful is not the list of pillars but the logic connecting them. Each pillar creates the conditions the next one requires: workforce development produces the capacity, institutional strengthening gives it authority and resources, governance and data standards direct how that capacity is used, and interoperable infrastructure ensures it operates within a connected, nationally owned system. When any one is missing, the system does not merely underperform: it generates a category of failure that investment in the remaining pillars cannot compensate for. That is the core analytical insight this paper advances.

Empirical validation is the essential next step. Longitudinal studies tracking the retention and system-level impact of health informatics fellowship graduates would test the core pillar 1–pillar 2 interdependency. Multicountry comparisons of divergent pillar-investment trajectories would assess whether coordinated multipillar investment produces better outcomes than single-pillar approaches. Implementation research should examine whether the readiness checklist in Table 2 predicts health informatics system performance across diverse country contexts.

Public health informatics in Africa is not merely a technical enterprise; it is the information infrastructure on which evidence-based health governance depends. African institutions that own their data, have the workforce to analyze them, and operate within governance frameworks that protect and direct their use are better positioned to set their own health priorities and hold their own systems accountable. The 4-pillar framework is offered as a practical tool for building that capacity systematically, equitably, and durably.

## Abbreviations

AES-PHI: Africa Epidemic Services-Public Health Informatics
AI: artificial intelligence
CDC: Centers for Disease Control and Prevention
DHIS2: District Health Information Software 2
HIS: health information system
NPHI: national public health institute
PHEOC: public health emergency operations center
WHO: World Health Organization
